# Neuroimaging in a memory assessment service: a completed audit cycle

**DOI:** 10.1192/pb.bp.113.043398

**Published:** 2014-02

**Authors:** Tarun Kuruvilla, Rui Zheng, Ben Soden, Sarah Greef, Iain Lyburn

**Affiliations:** 1 2gether NHS Foundation Trust, Cheltenham; 2 Stoke Road Surgery, Cheltenham; 3 Gloucestershire Hospitals NHS Foundation Trust, Cheltenham; 4 Cranfield University, Health Division

## Abstract

**Aims and method** A clinical audit was used to compare neuroimaging practice in a memory assessment service prior to and 6 months after implementation of guidance, developed from national and European guidelines and adapted to local resource availability, with multislice computed tomography (CT) as first-line structural imaging procedure.

**Results** Referrals to the service nearly doubled from the initial audit to the re-audit. Patients having at least one neuroimaging procedure increased from 68 to 76%. Patients with no reason documented for not having imaging significantly reduced from 50% to less than 1%. Despite the larger number of referrals, the mean waiting times for the scans only increased from 22 to 30 days. Variations in practice between the sectors reduced.

**Clinical implications** Disseminating evidence-based guidelines adapted to local resource availability appears to have standardised neuroimaging practice in a memory assessment service. Further research into the clinical and cost benefits of the increased scanning is planned.

It is estimated that 1 million people in the UK will be living with dementia by 2021, with current costs of dementia care to the economy being around £23 billion per year.^1^ The National Dementia Strategy highlighted the benefits of early, accurate diagnosis of dementia and its subtypes; not just for early access to disease-specific treatments, but because early diagnosis followed by psychosocial interventions significantly improves quality of life for patients and carers, thereby delaying the need for residential care.^2^ Yet only around 43% of people with dementia get a formal diagnosis,^1^ with wide regional variations. This led to the setting up of an All Party Parliamentary Enquiry Group to improve dementia diagnosis rates.

Neuroimaging is emerging as the most important ancillary investigation in the diagnostic work-up of dementia, with most clinical guidelines recommending at least one structural imaging procedure in every patient with suspected dementia.^3,4^ The traditional purpose of imaging was to exclude potentially treatable causes for cognitive impairment, such as tumours, haematomas and hydrocephalus. With advances in technology, neuroimaging is also used to include diagnosis of the dementia subtype.

In response to the National Dementia Strategy, we set up a county-wide memory assessment service with a well-defined assessment pathway for early diagnosis of dementia. It quickly became clear that there was wide variation in neuroimaging practice within the different sectors, establishing the need for clear, evidence-based clinical guidelines.

## Method

This clinical audit took place within the memory assessment service of ^2^gether NHS Foundation Trust for Gloucestershire, following approval from the Trust's audit department. The initial audit was carried out on all referrals to the service for the month of January 2010, 3 months after the service's inception. A data collection tool was developed, piloted and then used to collect information from patients' case notes. Local neuroimaging guidelines (Box 1) were then drawn up, after consultation with the service's old age psychiatrists and radiologists, based on the National Institute for Health and Care Excellence (NICE) and the European Federation of Neurological Societies (EFNS) guidelines,^4,5^ but adapted to local resource availability, namely increased availability of multislice computed tomography (CT) scans compared with magnetic resonance imaging (MRI). These guidelines were incorporated into the memory assessment service pathway in August 2010. They were included in the assessment pack, uploaded to the intranet and widely disseminated to memory assessment service clinicians at regular training sessions.

**Box 1** Neuroimaging guidelines for the ^2^gether NHS Foundation Trust memory assessment service

1. Structural neuroimaging should be used in the assessment of people with suspected dementia to exclude other cerebral pathologies and to help establish the subtype diagnosis.
2. A structural neuroimaging procedure, usually multislice computed tomography (CT) with coronal reformats, should be carried out at least once in every patient, unless there are good documented reasons for not doing so.
3. Hippocampal atrophy, for diagnosis of Alzheimer's disease, is best seen on magnetic resonance imaging (MRI).
4. Hippocampal atrophy may also be visualised in the coronal reformats of the modern multislice CT scanner.
5. In early onset Alzheimer's disease, MRI may be needed to localise atrophy to the more posterior regions.
6. MRI is more sensitive to subtle vascular changes and to changes that may indicate specific conditions such as frontotemporal dementia, multiple sclerosis, progressive supranuclear palsy, corticobasal degeneration and prion disease.
7. Functional neuroimaging with fluorodeoxyglucose-positron emission tomography (FDG-PET)/CT or hexamethylpropyleneamine oxime (HMPAO) SPECT may be used to help differentiate between Alzheimer's disease and frontotemporal dementia if the diagnosis is in doubt.
8. Functional neuroimaging with dopaminergic SPECT (DATScan™) may be used to help establish the diagnosis in those with suspected dementia with Lewy bodies if the diagnosis is in doubt.

The re-audit was carried out on all referrals to the service for the month of March 2011, 6 months after the implementation of the local guidelines. The same data collection tool was used but with one additional question, namely whether coronal reformats on CT scans were requested or not. By the time of the re-audit, all paper case notes had been replaced by electronic case notes. Data were collected and analysed using SPSS version 19 for Windows.

## Results

Table 1 shows that in the initial audit, for the month of January 2010, a total number of 89 patients were referred to the memory assessment service, for whom 82 case notes (92%) were identified and included in the audit. In the re-audit in March 2011, the memory assessment service referrals had nearly doubled to 150 patients, of which 140 (93%) were identified and audited. The patient characteristics appear to be similar in both audits despite the significant increase in numbers for the re-audit.

Table 2 shows that in the initial audit, 68% (56 out of 82) of the included patients had at least one neuroimaging procedure done, either referred through the memory assessment service pathway or done prior to the referral to the service. In the re-audit this percentage had increased to 76% (106 out of 140) of the included patients. However, this improvement was not statistically significant (*P* = 0.23; χ^2^ = 1.44; d.f. = 1). The proportion of the neuroimaging ordered through the service also marginally improved from 59 to 63%.

The case notes of those who had no neuroimaging performed were scrutinised and we found that in the initial audit, 50% (13 out of 26) had no reason documented for not having neuroimaging. In the re-audit this had significantly reduced to only 1 out of 34, or less than 1% (*P*<0.001; χ^2^ = 8.24; d.f. = 1). In the re-audit, 45 out of 78 (58%) of CT head scans requested through the service had coronal reformatting done. This information was not available for the initial audit.

We looked at the waiting times from scan request to scan appointments. The mean wait was 30 days (range 11–71) in the re-audit compared with 22 days (range 6–42) in the initial audit. It was interesting to note that despite the significant increase in the number of scan requests, the waiting times had only marginally increased.

We also compared the percentage of patients who underwent neuroimaging in each sector across the county and found that the gap between the best and worst performing sectors had narrowed in the re-audit, suggesting increased uniformity of practice across the county.

## Discussion

The traditional purpose of neuroimaging in dementia is to exclude potentially treatable causes for cognitive impairment, with CT and MRI performing equally well in this regard.^5^ The yield for this purpose varies between 1 and 10% and may be even lower.^6,7^ However, Gifford *et al*^8^ showed that the there is considerable uncertainty in the evidence behind clinical prediction rules to identify which patients with dementia should undergo neuroimaging, and the application of these rules may miss patients with potentially reversible conditions, hence it is widely accepted that a structural imaging procedure should be performed routinely in each patient with suspected dementia.^8^

With recent technological advances, neuroimaging is also now used to include diagnosis of the four common dementia subtypes. Hippocampal atrophy has emerged as a sensitive and specific marker for Alzheimer's disease,^9,10^ and is recognised as a biomarker of neuronal injury in the updated National Institute on Aging and the Alzheimer's Association (NIA–AA) workgroup's diagnostic criteria.^11^ The overall sensitivity and specificity of hippocampal atrophy for detecting mild to moderate Alzheimer's disease *v*. controls was 85% and 88% in a meta-analysis.^12^ In the widely used National Institute for Neurological Disorders and Stroke and the Association Internationale pour la Recherché et l'Enseignement en Neurosciences (NINDS–AIREN) diagnostic criteria for vascular dementia, structural neuroimaging is essential for the diagnosis by demonstrating a link between dementia and cerebrovascular disease in the form of: large vessel infarcts; single, strategically placed infarcts; multiple basal ganglia and white matter lacunae; or extensive periventricular and deep white matter ischaemia.^13^ Dementia with Lewy bodies may not have diagnostic structural imaging changes but functional imaging with dopaminergic single-photon emission computed tomography (SPECT), also called the DATScan™, is useful to differentiate dementia with Lewy bodies from Alzheimer's disease with

**Table 1:** Patients' characteristics

Patient characteristics	2010 audit	2011 re-audit
Total referrals to MAS for the calendar month, *n*	89	150
Case notes included in audit, *n/N* (%)	82/89 (92)	140/150 (93)
Gender, *n* (%)		
Male	31 (38)	60 (43)
Female	51 (62)	80 (57)
Age, years: *n* (%)		
<65	5 (6)	14 (10)
65-79	23 (28)	43 (31)
≥80	54 (66)	83 (59)
Duration of cognitive symptoms, years: *n* (%)		
<1	15 (18)	39 (28)
1-5	56 (68)	62 (44)
6-10	5 (6)	4 (3)
≥10	0 (0)	2 (1)
Not documented	6 (7)	33 (24)
History of vascular disease/risk factors: cerebrovascular disease, cardiac disease, peripheral vascular disease, vascular risk factors, *n* (%)	59 (72)	100 (71)
Atypical features: previous cranial pathology, head injury, raised intracranial tension, features of normal pressure hydrocephalus, focal neurology on examination, *n* (%)	16 (20)	13 (9)
MMSE score, *n* (%)		
<10 (severe)	0 (0)	7 (5)
10-20 (moderate)	32 (39)	37 (26)
21-26 (mild)	25 (30)	36 (26)
>26 (normal)	15 (18)	30 (21)
Other (declined, unable to do, unrecorded)	10 (12)	30 (21)
Final diagnosis, *n* (%)		
Mild cognitive impairment	10 (12)	15 (11)
Alzheimer's disease (including mixed dementia)	18 (22)	59 (42)
Vascular dementia	12 (15)	12 (8)
Dementia with Lewy bodies	1 (1)	1 (1)
Frontotemporal dementia	0 (0)	2 (1)
Other diagnosis (depression, brain tumour, multiple sclerosis, alcohol-related cognitive impairment, no dementia	8 (10)	12 (9)
No diagnosis recorded (awaiting diagnosis, declined, died, moved away)	33 (40)	39 (28)

MAS, memory assessment service; MMSE, mini-Mental State Examination.

**Table 2:** Neuroimaging performed

	2010 audit *n* = 82	2011 re-audit *n* = 140
Patients who had at least one neuroimaging procedure (through MAS or previous), *n* (%)	56 (68)	106 (76)
Neuroimaging requested through MAS, *n* (%)	48 (59)	88 (63)
Previous neuroimaging, *n* (%)	8 (9)	18 (13)
Patients who declined assessment/scan, *n* (%)	13 (16)	29 (21)
Patients with documented reasons for not having neuroimaging (unable to tolerate scan, moved out of area, died), *n* (%)	0 (0)	4 (3)
Patients with no reason documented for not having neuroimaging, *n* (%)	13 (16)	1 (1)
Of the patients who did not have any neuroimaging, proportion with no reason documented, *n/N* (%)	13/26 (50)	1/34 (3)
Type of neuroimaging performed (through MAS and previous), *n/N* (%)		
CT scan	49/56 (88)	92/106 (87)
MRI scan	5/56 (9)	14/106 (13)
Other (FDG-PET/CT, HMPAO-SPECT)	2/56 (4)	0/106 (0)
Coronal imaging on CT scans requested through MAS, *n/N* (%)	Data not available	45/78 (58)
Length of time between scan requests and scan appointments, days		
Range	6-42	11-71
Mean	22	30
Range across different sectors of patients who had a neuroimaging procedure, %	33-100	58-84

CT, computed tomography; FDG-PET, fluorodeoxyglucose-positron emission tomography; HMPAO-SPECT, hexamethylpropyleneamine oxime single-photon emission computed tomography; MAS, memory assessment service; MRI, magnetic resonance imaging.

**Fig 1:**
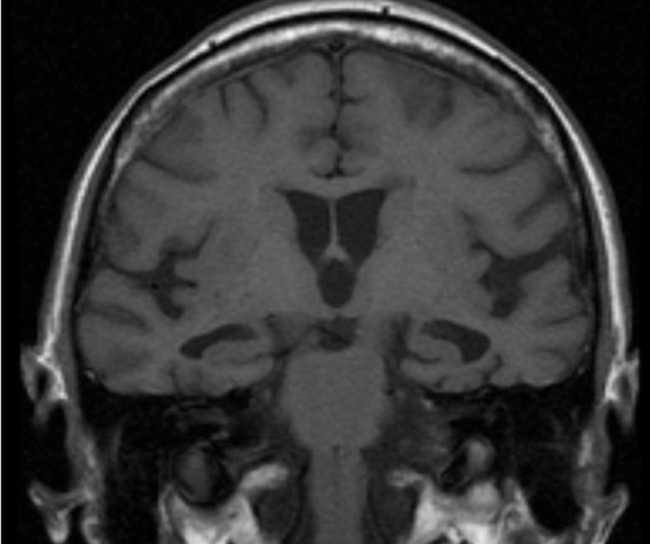
Magnetic resonance imaging: T_1_-weighted coronal image showing bilateral hippocampal atrophy in Alzheimer's disease.

sensitivity and specificity of around 85%^14^ (low dopamine transporter uptake on the DATScan™ is also included as a suggestive feature in the Third Dementia with Lewy Bodies Consortium consensus diagnostic criteria).^15^ As per the new International Consensus Criteria for behavioural variant frontotemporal dementia, probable behavioural variant frontotemporal dementia can only be diagnosed if, in addition to the clinical findings, there are imaging correlates of at least one of frontal and/or anterior temporal lobe atrophy on MRI/CT, or frontal and/or anterior temporal hypoperfusion or hypometabolism on hexamethylpropyleneamine oxime (HMPAO) SPECT or fluorodeoxyglucose-positron emission tomography (FDG-PET).^16^

There is no clear guidance on whether CT or MRI should be the first-line structural imaging procedure. Although they are equally good in excluding potentially treatable conditions, MRI is more sensitive to subtle vascular changes such as strategic infarcts, and to changes indicative of specific conditions such as multiple sclerosis, prion disease, the Parkinson's plus syndromes and frontotemporal lobar degeneration.^12,17^ Patients with early-onset Alzheimer's disease often have non-amnesic presentations and here MRI may be needed to localise atrophy to the more posterior regions of the precuneus and posterior cingulate cortex.^18^ However, CT may be more widely available and cheaper to access than MRI, and the modern multislice CT has shown excellent reliability, compared with MRI, for detecting hippocampal atrophy (Fig. 1 and Fig. 2), global cortical atrophy and white matter changes.^19^ In our county, as in most regions, multislice CT was more readily accessible and cheaper than MRI, hence multislice CT with coronal reformats (to look for hippocampal atrophy) was recommended as the first-line structural imaging procedure, with MRI reserved for certain specific indications.

This completed audit showed that since its establishment, there has been a significant increase in referrals to our memory assessment service, which could be due to increased awareness of dementia among the general public and in primary care as a result of government campaigns. It

**Fig 2:**
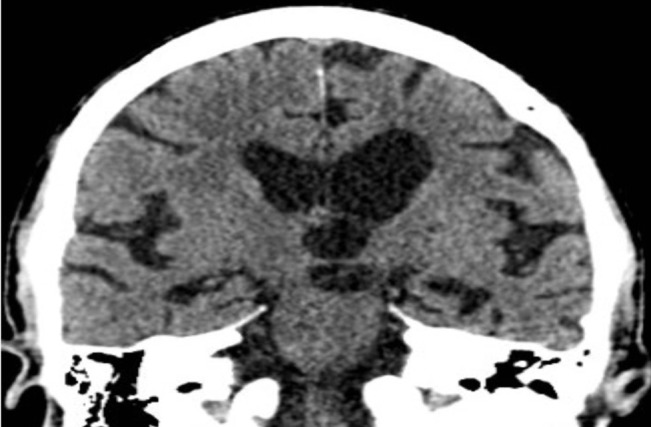
Multislice computed tomography with reconstruction in the coronal plane showing bilateral hippocampal atrophy in Alzheimer's disease.

is hoped that this would lead to increased diagnostic rates and earlier diagnoses of dementia.

Developing and disseminating evidence-based guidelines derived from national and international recommendations but adapted to local resource availability seems to have improved imaging practice. In the re-audit, our primary outcome of the total percentage of patients having imaging increased by 8%. Although this was not statistically significant, the sample size may not have been large enough, resulting in a type II error. The proportion of patients for whom there was no reason documented for not having imaging had significantly reduced. This is important because there are going to be other reasons for not having imaging, such as patient refusal and moving out of area, which are beyond the control of the clinicians. Another key outcome measure was that the variations in referral rates between the different sectors had reduced, suggesting standardisation of practice. In the re-audit, the majority of CT scans requested through the memory assessment service had coronal reformats done to look for hippocampal atrophy. Magnetic resonance imaging and functional scan numbers were too small to allow for any meaningful analysis on the appropriateness of the requests.

Our memory assessment service accepts referrals for patients with mild to moderate cognitive impairment suspected to be due to dementia. Patients with severe cognitive impairment are diagnosed in primary care with assistance from community dementia nurses. It is argued that here neuroimaging may not be beneficial, especially if it is not going to influence management plans. As per our service pathway, patients are initially seen by a nurse for a core assessment, findings of which are discussed at a multidisciplinary meeting where further assessments, including neuroimaging, are requested. After all assessments are complete, patients are seen in memory clinics for diagnosis. The guidelines mandate neuroimaging for people with suspected dementia so we would have liked to exclude the ‘worried well’ and patients with clear mild cognitive impairment from the data analysis, but our clinical pathway did not allow this and the scan result may have also influenced the final diagnosis in these two groups.

A limitation of this study, as with all retrospective case-note-based studies, is that the data gathered are only as good as what is entered in the notes.

It is beyond the remit of this clinical audit to say whether the increased imaging improved the accuracy of the diagnosis of dementia or to look at its cost-effectiveness, although with the modern multislice CT machines, scanning times and costs have significantly reduced. As per the National Schedule of Reference Costs for NHS Trusts,^20^ a CT brain scan without contrast has a unit cost of about £100 and an MRI brain scan without contrast has a unit cost of about £175. In our audit, despite the doubling of scan requests, the waiting times for a scan had not significantly increased. More research is planned to evaluate the clinical and cost-effectiveness of the increased scanning and to look at the quality of scan reporting. The part-Medical Research Council-funded evaluation of the brain health centres, a nurse-led service using computerised cognitive tests and computerised reporting of MRI scans, for timely dementia diagnosis in primary care is awaited.^21^ In our service, routine inspection of scan images on the computerised picture archiving and communications system (PACS) and improved communication with the radiologists have enabled old age psychiatrists to gain some expertise in reading scans, and this has also allowed the use of the images for patient education purposes. Other memory services may want to use our evidence-based neuroimaging guidelines to help standardise practice.
